# Brief, pragmatic measure of emotion dysregulation in young people – a preliminary validation of the BER-5

**DOI:** 10.1186/s40479-025-00285-4

**Published:** 2025-03-13

**Authors:** Iselin Solerød Dibaj, Sudan Prasad Neupane, Lars Mehlum

**Affiliations:** National Centre for Suicide Research and Prevention, University of Oslo, Oslo, Norway

**Keywords:** Emotion dysregulation, Borderline personality disorder, Clinical screening

## Abstract

**Background:**

Emotion dysregulation is a transdiagnostic construct associated with mental health problems, including self-harm and borderline personality disorder (BPD). Although often targeted in clinical practice, the majority of psychometric assessment instruments of emotion dysregulation are developed for research purposes, and there is a need for an adapted version to be used in a clinical screening setting. The main aim of this study was to examine psychometric properties of a brief, pragmatic measure of emotion dysregulation, the Brief Emotion Regulation Scale − 5 items (BER-5).

**Methods:**

The sample consisted of 60 young adults (mean age 28.1 years) who participated in a 12.4 years follow-up study of an RCT of Dialectical Behaviour Therapy’s long-term effect. Cronbach’s alpha was used to calculate internal consistency. Concurrent and convergent validity were examined using Spearman’s correlation in comparison with other measures, and logistic regression as well as area under the curve to examine its ability in terms of differentiating between BPD diagnosis and trait levels. Optimal cut-off points were explored using Receiver Operating Curves.

**Results:**

Our results indicated adequate internal consistency both in adolescence and in young adulthood, as well as high convergence with a gold-standard measure of emotion dysregulation, and moderate convergence with symptom measures of anxiety and depression. BER-5 was able to differentiate between participants with no BPD from subthreshold or full BPD diagnosis in adulthood, and a cut-off score of 5 was found optimal in terms of sensitivity and specificity in identifying individuals with BPD.

**Conclusions:**

The BER-5 is a brief, pragmatic measure of emotion dysregulation with good psychometric properties and is potentially a useful screening tool for clinicians working in specialized health care settings.

**Supplementary Information:**

The online version contains supplementary material available at 10.1186/s40479-025-00285-4.

## Introduction

Mental health problems and self-harm frequently have their onset during adolescence [1]. Studies have shown that mental ill-health and rates of self-harm are increasing in this age group [2, 3] and, correspondingly, presentations by adolescents to emergency care for self-harm episodes have increased strongly. The reasons for these developments are poorly understood [4], however, difficulties with emotion regulation, or emotion dysregulation, seems to be a common pathway through which mental ill-health and self-harm is frequently linked [5]. Indeed, adolescents who struggle to adequately regulate emotional states, are at higher risk not only for mental disorders and self-harm, but of substance abuse and a range of other risky behaviours [6], in addition to having poorer academic and interpersonal functioning [7]. Similar associations have been reported in adult clinical populations [8], and although self-harm tends to decrease from adolescence to adulthood [9], underlying problems with emotion dysregulation might persist [10].

In a situation with rising rates of adolescent mental ill-health and self-harm prevalence, timely detection and intervention are key to prevention of severe outcomes such as chronic psychiatric disorders and suicide. Patients with self-harm might require different interventions if they also present with persistent emotion dysregulation problems [11]. Effective interventions for adults and adolescents with self-harming behaviours and emotion dysregulation developed and empirically supported over the last 1–2 decades, such as Dialectical Behaviour Therapy, have become more available in many countries [12, 13]. For the purposes of early identification of patients in need of such interventions, time-effective, valid and precise screening methods for emotion dysregulation should be a high priority. This may be challenging for clinicians due to time constraints and limited resources, thus there is a need for more efficient and pragmatic screening measures.

Regarded the “gold-standard” by many for measuring emotion dysregulation, the *Difficulties in Emotion Regulation Scale DERS-36* [14] is a 36-item self-report questionnaire with good psychometric properties, but it has largely been used in a research context. In a clinical context, however, administering and scoring this lengthy instrument might not be feasible. Consequently, several shorter versions of the DERS with decent psychometric properties and high concordance with the original scale, have been proposed [15]. As with all self-report measures, these scales carry risk of reporting bias, which may be a prominent problem with measuring emotion regulation capacity in the case of emotionally dysregulated individuals where their emotional state in the moment of reporting may fluctuate strongly and yield results that may be less representative of their average score [16]. In clinical practice, self-report measures are often supplemented by interview data that allow clinicians to elicit or interpret the symptoms more carefully. Evaluation of emotion dysregulation is often part of personality disorder assessment, such as the SCID-II interview [17]. However, this is a time-consuming interview and, arguably, a more pragmatic approach might be warranted. For that reason, we have constructed a short, composite measure combining self-report and clinician rated variables that could potentially serve as a more efficient, pragmatic and valid measure for screening of emotion dysregulation in adolescents. Previously, this measure has been used to distinguish between suicide attempters and non-attempters in a sample of treatment-seeking adolescents with BPD traits and repeated self-harm [18], as well as to examine trajectories of change in emotion dysregulation among adolescents [19]. The internal consistency of the composite measure has been reported as good (α = 0.72) [18], however, its validity has not yet been tested. In this study, we, thus, aimed to examine the psychometric properties of this brief, pragmatic measure (henceforth referred to as the BER-5 (Brief Emotion Regulation-5 items) of emotion dysregulation in terms of concurrent and convergent validity. Finally, we aimed to determine a suitable cut-off point to putatively classify individuals with borderline personality disorder (BPD).

## Methods

### Participants and procedures

Participants were recruited from child and adolescent psychiatric outpatient clinics in Oslo as part of an RCT comparing Dialectical Behavior Therapy and Enhanced Usual Care for adolescents with recurrent self-harm and BPD features. The main results from this trial have been published elsewhere [20, 21], but in this context we have utilized the data set to investigate potential new and simpler ways of measuring emotion dysregulation. Inclusion criteria for the RCT were a history of ≥ 2 episodes of self-harm, whereof ≥ 1 within the last 16 weeks, ≥ 3 criteria of DSM-IV BPD (including the self-destructive criterion) *or* ≥ 2 criteria of DSM-IV BPD *plus* ≥ 2 subthreshold-lever criteria, and Norwegian fluency. Exclusion criteria were a diagnosis of Bipolar I disorder, any psychotic disorder, intellectual disability or Asperger’s syndrome. Altogether 294 patients were screened for eligibility, 97 went through clinical assessment and the final sample consisted of 77 adolescents, aged 12–18 years. For the current validation study, the main part of the data was included from the final follow-up assessment 12.4 years post randomization. To compute and compare internal consistency across time points, we used data from assessments at baseline, 3.1 and 12.4- years post randomization.

### Measures

Our main variable of interest in this study is a composite variable for emotion dysregulation, encompassing both self-report as well as clinician-rated items. The composite variable was generated as a sum score (with a range of 0–10) of the following equally weighted items: Two items from the Youth Self Report Scale [22]; “sudden mood swings” and “intense anger”, one item from the Borderline Symptom List-23 [23]; “frequent changes in the mood between anxiety, anger and depression”, and two Borderline Personality Disorder criteria assessed through the Structured Clinical Interview for DSM-IV [17]; “affective instability” and “inappropriate anger”. The operationalizations of variables as well as a syntax (for STATA v. 17) are provided in the supplementary materials. The selection of items for the scale was not grounded in empirical evidence or predetermined theoretical frameworks. Instead, we selected items from our variable set to cover putatively varying aspects of emotion dysregulation from a clinical perspective.

To test concurrent validity, we used a short version of the Difficulties in Emotion Regulation Scale (DERS-16; [24]). This is a 16-item self-report questionnaire (each item scored from 1 to 5). The DERS-16 has demonstrated excellent internal consistency (*α* = 0.92*)* and a strong association with the original DERS-36 (*r* =.93). To test convergent validity, we used the following variables: Symptom Check List 6 (SCL-6) as a self-report measure of anxiety symptoms, Montgomery and Aasberg Depression Rating Scale (MADRS; [25]) and Moods and Feelings Questionnaire (MFQ; [26]) as clinician and self-rated measures of depressive symptoms and Suicidal Ideation Questionnaire for youth (SIQ-Jr; [27]) as a self-report measure of suicidal ideation. These types of symptoms are highly prevalent both in adolescents and adults with self-harm behaviour and are associated with emotion dysregulation, yet they represent different dimensions and constructs and a measure of emotion dysregulation should have psychometric properties enabling it to reflect this. Thus, we would expect these variables to be related to the BER-5, however to a lesser extent than the DERS-16. To examine the BER-5’s ability to differentiate between known diagnostic groups, we use the diagnosis of BPD, as evaluated by the research clinician (based on the SCID-II diagnostic interview) and levels of BPD traits, as a valid measure of emotion dysregulation should be associated with BPD, and more so with higher number of traits. Although the BER-5 is intended as a measure of emotion dysregulation, and not BPD per se, one would expect that a valid measure of emotion dysregulation should be able to distinguish between participants with and without BPD.

### Data analysis

To examine concurrent and construct validity, Spearman correlation (rho, ρ) was calculated. The Shapiro-Wilk test was used to test for normality. Factorial validity was examined using principal component analysis and confirmatory factor analysis. We tested whether the BER-5 scores at different time points could predict concurrent and prospective BPD diagnosis using binary logistic regression. To examine whether BER-5 outperformed more generic instruments in predicting BPD, the same analyses were performed for SCL-6 scores at baseline and the final follow-up. In addition, we compared it to the DERS-16 at the final follow-up. Receiver Operating Characteristic curves and the corresponding Area Under the Curve (AUC) were calculated for BER-5 compared to DERS-16 total score dichotomized into high vs. low scores, to differentiate between participants with and without BPD. Since subthreshold (3–4 BPD traits) and full-syndrome BPD might be dimensionally rather than categorically different clinical presentations [11] we additionally performed similar analyses to differentiate between participants with above and below (0–2 traits) subthreshold BPD to find the optimal cut-off point of DERS-16 in the present sample. Subsequently, ROC analysis was used to determine the suitable cut-off for the BER-5 in order to classify the dichotomized groups. As we only had DERS-16 data for the 12.4 year follow-up, we were unable to perform these analyses using the baseline data. However, we did investigate internal consistency across all time points, as well as examine BER-5’s ability to differentiate between participants with and without a BPD diagnosis at baseline. The correlation and regression analyses were conducted in STATA 17.0, while the ROC analysis, principal component analysis and factor analysis was performed in SPSS v. 29.

## Results

The sample consisted of 77 adolescents (88% female; mean age 15.6) at baseline, of which 60 were re-assessed as young adults (87% female; mean age 28.1) at 12.4 years follow-up. At the 12.4 years follow-up, the overall sample had a mean BER-5 score of 3.25 (SD = 2.79); participants with a BPD diagnosis (5 or more traits) scored 6.75 (SD = 1.58); those with subthreshold BPD (3–4 traits) scored 7.5 (SD: 0.71), and those without BPD (0–2 traits) scored 2.52 (SD: 2.39). The corresponding DERS-16 scores were 41.9 (SD: 15.27) in the overall sample; 62.9 (SD: 10.6) in those with BPD, 60.0 (SD: 4.2) in those with subthreshold BPD; and 37.82 (SD: 12.73) in participants without BPD.

### Reliability

Skewness of the BER-5 was 0.1846 and kurtosis was 0.0004. Internal consistency was acceptable, with a Cronbach’s alpha of 0.72 at baseline, 0.69 at 3.1 years follow-up, and 0.83 at 12.4 years follow-up. Inter-item correlations of the BER-5 for the final time point are displayed in Table 1. All items were positively and significantly inter-correlated except for the correlation between items 4 and 5 (ρ= 0.21). Inter-item correlations at baseline and at 3.1 years follow-up were weaker though mostly statistically significant. The correlation matrix for the final follow-up is presented in Table 1, whereas correlation matrices for the other timepoints are included in the supplementary materials (Table S2).

**Table 1:** Inter-item correlations between BER-5 items at the 12.4 years follow-up. Reported values are spearman correlation coefficient Rho (ρ)

12.4 years	Q1_12	Q2_12	Q3_12	Q4_12	Q5_12
Q1_12	1.00				
Q2_12	0.62*	1.00			
Q3_12	0.66*	0.46*	1.00		
Q4_12	0.45*	0.67*	0.48*	1.00	
Q5_12	0.59*	0.34*	0.56*	0.21	1.00

* *P* < .05; ** *P* < .001

### Factorial validity

**Fig. 1:**
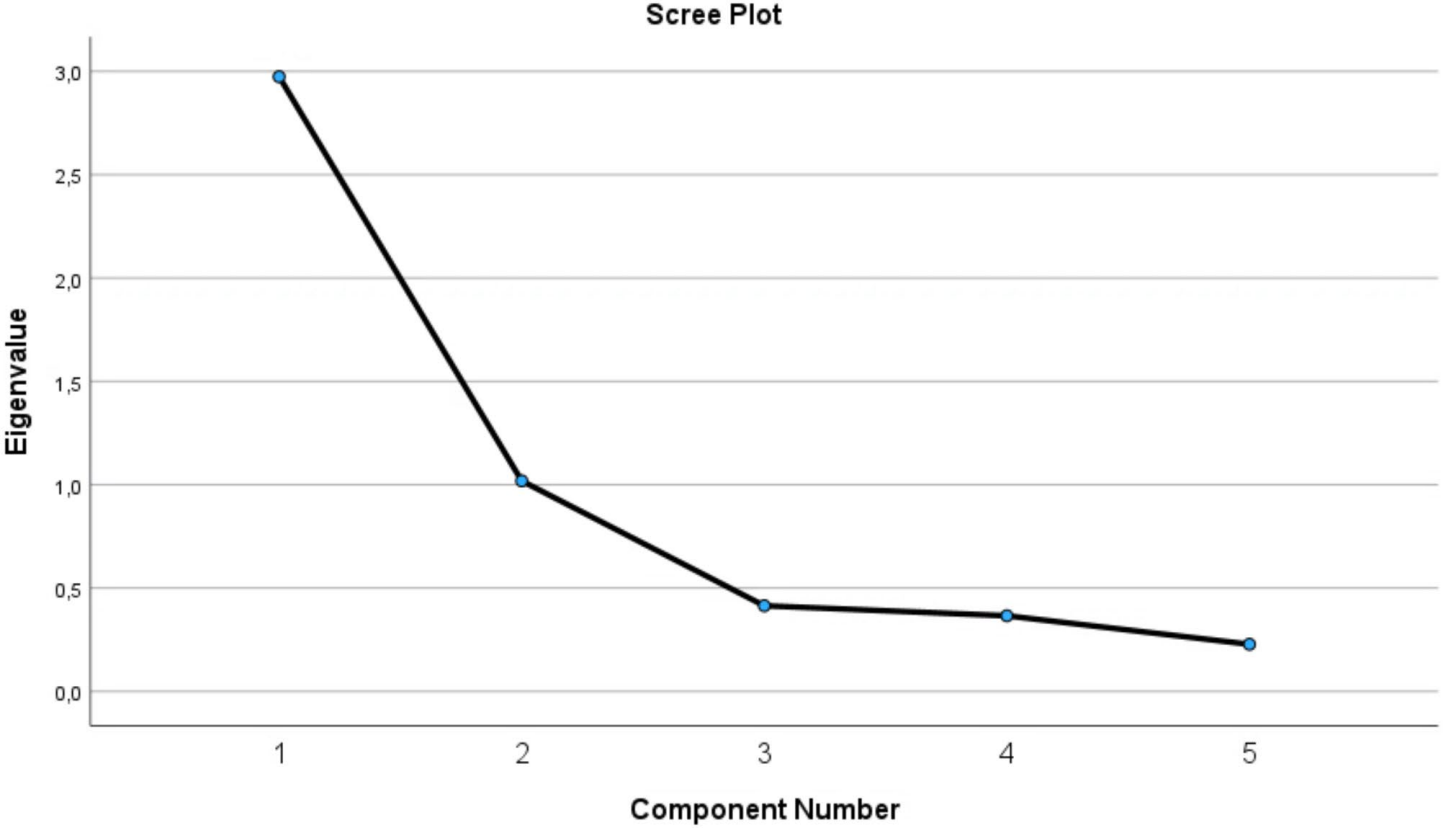
Scree plot of Eigen values from the exploratory factor analysis of the BER-5

All factors were above 4 in the commonality test in the principal component analysis, and suggestive of a one-component solution (see Table S1 in supplementary materials). The first item was the most strongly related to this component (0.87), while the others followed in descending order with the lowest being 0.66. In the confirmatory factor analysis, we found support for a 1-factor structure, explaining 59.47% of the variance. The Kaiser-Meyer Olkin Measure of sampling adequacy was deemed adequate (0.736) and Bartlett’s test of sphericity was statistically significant ($\:{\chi\:}^{2}$= 127.61, *p* < .001). A Scree plot is provided in Fig. 1 and the covariance matrix and Eigenvalues from the principal component analysis are reported in the supplementary materials.

### Concurrent and convergent validity

**Fig. 2:**
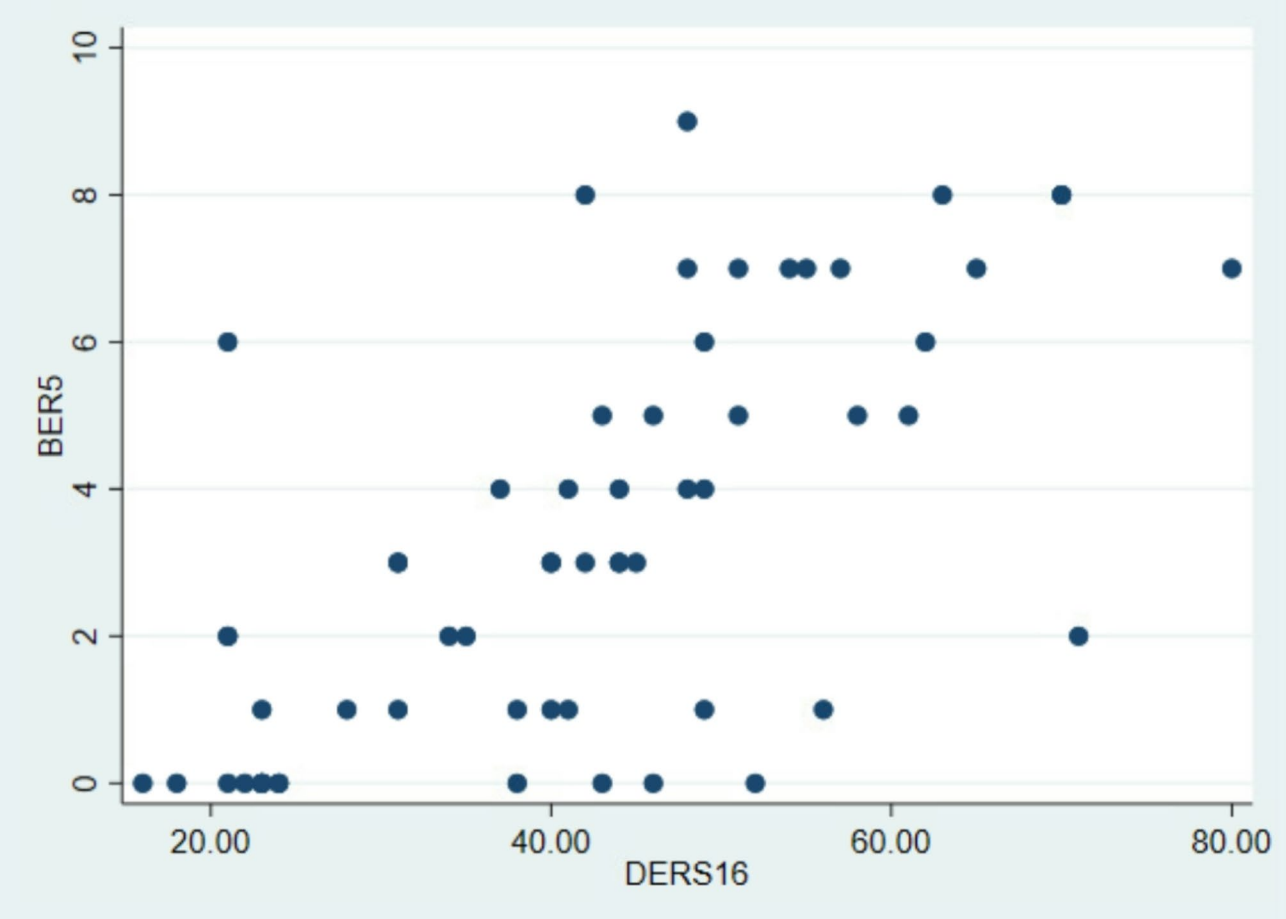
Scatterplot over associations between BER-5 and DERS-16 scores

The BER-5 and DERS-16 scores were positively inter-correlated (ρ = 0.66, *p* = < 0.0001, see Fig. 2), indicating moderate-to-high concurrent criterion validity. The BER-5 showed moderate, positive associations with SCL-6 (ρ = 0.47, *p* = .0002), SMFQ (ρ = 0.39, p.0019), SIQ-JR (ρ = 0.48, *p* = .0002), and MADRS (ρ = 0.51, *p*<-.000), indicating convergent validity.

### Differentiation by known groups

The logistic regression analyses indicated time-dependent associations between the BER-5 and a BPD diagnosis. At baseline, higher BER-5 values predicted higher odds of having a concurrent BPD diagnosis (OR: 2.24 [95% CI: 1.38–3.63], *p* = .001). At 12.4 years follow-up, higher BER-5 scores were associated with higher odds of having a BPD diagnosis (OR: 2.02 [95% CI: 1.26–3.23], *p* = .003). Longitudinally, higher BER-5 values measured at baseline and follow-up at 3.1 years did not predict having a BPD diagnosis in adulthood (OR: 1.29 [95% CI: 0.93–1.78], *p* =.12 and OR: 0.98 [95% CI: 0.73–1.32], *p* = .91, respectively).

To examine whether the BER-5 outperformed more generic screening measures in predicting concurrent BPD, the same analysis was conducted for SCL-6. SCL-6 did differentiate between participants with and without a BPD diagnosis at both baseline (OR: 1.18 [95% CI:1.01–1.39], *p* = .04) and the 12.4 years follow-up (OR: 1.27 ]95% CI: 1.04–1.53], *p* = .02). At the 12.4 years follow-up, the BER-5 also outperformed the DERS-16 (OR: 1.18 [95% CI:1.06–1.31], *p* = .002) in predicting a concurrent BPD diagnosis. Post-hoc analyses revealed that diagnostic variables such as current major depressive episode (t=−2.39, *p* = .02) or fulfilling criteria for a BPD diagnosis (F = 5.06, *p* =.0001) were associated with mean differences in both emotion dysregulation measures (data not shown for DERS-16).

### ROC analyses and area under the curve

**Fig. 3:**
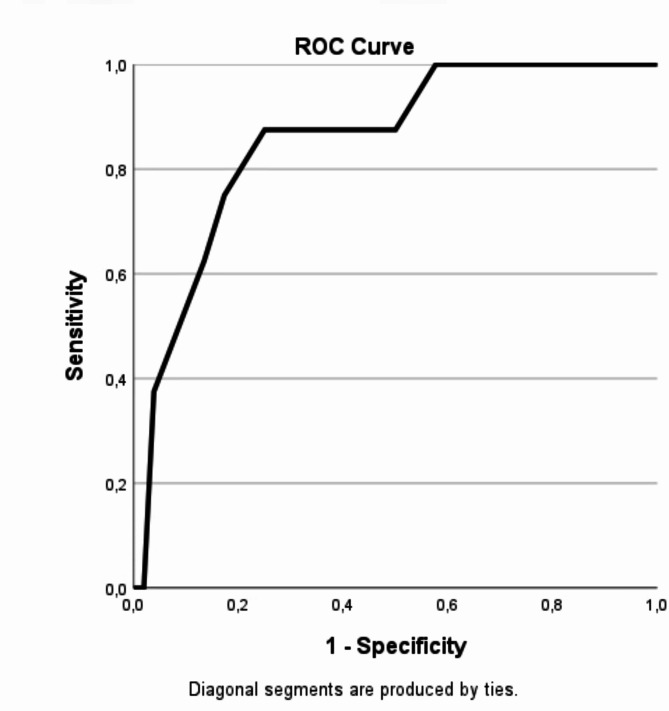
ROC analysis of DERS-16 dichotomized at 60, a cut-off that indicated subthreshold BPD or BPD diagnosis against BER-5

First, we ran ROC analysis of DERS-16 against BPD to find a suitable cut-off level for the DERS-16 that we could subsequently use to validate the BER-5. The AUC reached 0.927 and cut-off points at 50, 57.5 and 60 were deemed appropriate as potential cut-off points as these cut-off values corresponded to higher levels of sensitivity and specificity in the DERS-16’s ability to correctly identify participants with a BPD diagnosis, wherein the lowest (50) also included those with subthreshold BPD (see Table S5 in supplementary materials for details). Then, we dichotomized the DERS-16 scores into high and low, according to the three different cut-off points and conducted ROC analyses using these indicators to compare with the BER-5. The cut-off score at 60 yielded the highest AUC at 0.855 (SE: 0.067, see Fig. 3); all corresponding sensitivity and specificity values are presented in Table 2. At a BER-5 average score of 5, the sensitivity reached 87.5%; specificity reached 75%, while correctly classifying 77% cases and with positive and negative likelihood ratios of 3.5 and 0.17 respectively. Scoring above 5 in BER-5 was strongly associated with fulfilling diagnostic criteria for BPD ($\chi^2$ (1, *N* = 60) = 18.46, *p* < .001).

**Table 2:** Sensitivity and specificity values for ROC analysis of DERS-16 +/- 60 and BER-5

Cut-off point	Sensitivity	Specificity	Correctly classified	LR +	LR -
>=0	100%	0%	13.33%	1.00	-
>=1	100%	26.92%	36.67%	1.37	0
>=2	100%	42.31%	50%	1.73	0
>=3	87.5%	50%	55%	1.75	0.25
>=4	87.5%	65.38%	68.33%	2.53	0.19
**>=5**	**87.5%**	**75%**	**76.67%**	**3.50**	**0.17**
>=6	75%	82.69%	81.67%	4.33	0.30
>=7	62.5%	86.54%	83.33%	4.64	0.43
>=8	37.5%	96.15%	88.33%	9.75	0.65
>=9	0%	98.08%	85%	0	1.02
> 9	0%	100%	86.67%	-	1

With a cut-off point at 50, the AUC was 0.817 (SE: 0.06) and a cut-off point on the BER-5 indicated a sensitivity of 81.25% and specificity of 84%. The cut-off at > 57 yielded an AUC of 0.84, a sensitivity of 75% and specificity at 94%. All three ROC analyses supported an optimal BER-5 cut-off at 4.5 or 5. ROC curves (Figures S1−2) and sensitivity and specificity values for BER-5 cut-off points of 50 and 57.5 (Tables S3-4) are presented in the supplementary materials. We considered the optimal balance between high sensitivity and specificity, favouring sensitivity slightly as the BER-5 is intended as a screening instrument in a clinical setting where risk of adverse effects of false positives is low. Thus, an average value of BER-5 at 5 was considered an acceptable and pragmatic cut-off level.

## Discussion

We found that our novel, brief and pragmatic measure of emotion dysregulation (BER-5) had adequate psychometric properties, with adequate internal consistency both when measured in adolescence and young adulthood and high concurrent criterion validity when compared to the gold-standard measure of emotion dysregulation (DERS-16), suggestive of high convergent construct validity. Correlations with standard measures of anxiety and depression were weaker compared to the DERS, indicating acceptable divergent validity, suggesting that the BER-5 is not merely a measure of general distress. Next, the BER-5 was able to adequately differentiate between important clinical groups in the population of patients with varying levels of emotion dysregulation, particularly between participants with either full scale or subthreshold BPD from those with none or few BPD features. Moreover, the BER-5 outperformed both the SCL-6 and the DERS-16 in distinguishing participants with and without a BPD diagnosis. Finally, when applying a cut-off score of 5, the BER-5 had a satisfactory combination of sensitivity and specificity when it comes to identifying individuals with probable BPD.

Although the BER-5 was able to differentiate participants with or without a BPD diagnosis, it did not distinguish between those with subthreshold BPD compared to a full BPD diagnosis. Surprisingly, BER-5 scores were slightly higher in the subthreshold group compared to those fulfilling criteria for a full diagnosis. This may seem contradictory as emotion dysregulation is a core feature of BPD, and a more linear relationship between these variables would be expected, as more severe emotion dysregulation has been found to be associated with higher number of BPD traits [11]. Considering the content of the scale, one possible explanation is that the BER-5 taps more into the *negative affectivity* trait of BPD, and less so into the other trait domains associated with the disorder, such as antagonism, detachment, disinhibition or psychoticism [28]. Moreover, both negative affectivity and emotion dysregulation are associated with several other mental health disorders other than BPD, including other personality disorders, which we did not assess in this study.

In patient screening in clinical settings, time-efficiency and heuristics to aid decision making is highly important. The suggested cut-off point of 5 was able to differentiate between patients with or without either subthreshold or a full diagnosis of BPD, using scores from only five items. As mentioned above, a measure of emotion dysregulation should ideally contain both self-report and clinician-rated sources of information since the nature of emotion dysregulation may severely bias purely self-report accounts of this important clinical feature [16]. In many clinical contexts, it is unrealistic or even unfeasible to use both lengthy interviews and questionnaires developed for these purposes, thus the BER-5 may be a useful tool for assessing emotion dysregulation capturing both sources of information. To our knowledge, this is the shortest measure of emotion dysregulation that shows good psychometric properties comparable to shorter versions of DERS [15]. Our pragmatic measure carries the advantage of utilizing variables and data that many clinics are already collecting routinely. Thus, as a pragmatic measure in a clinical setting, the BER-5 might be a helpful tool for clinicians in quickly, but still reliably and validly, screening patients for emotion dysregulation.

### Limitations and future directions

The current study benefits from being based on a clinically relevant sample collected in a specialized health care context, and with access to both self-reported as well as stringent clinician-rated data. This allowed us to examine psychometric properties of the BER-5 within the same population as it is intended for. However, in addition to the modest sample size, a great limitation of this work is its post-hoc nature; thus it is not validated in a normative sample nor systematically developed a priori. Moreover, data allowing a study of the convergent validity in relation to the DERS-16 was only available for the adult follow-up, and whether the findings are generalizable to other age and gender groups needs to be investigated in future studies. Also, all correlations were cross-sectional, thus the predictive validity of the BER-5 should be examined in a more suitable design with shorter time intervals. As we used data from an RCT, where one of the interventions previously has been shown to be associated with improvement on the BER-5 [19], a rigorous examination of predictive validity was compromised. Moreover, since our results were based on a sample of adolescents/young adults, who were predominantly female, in a narrow geographical context and participating in an RCT comparing DBT-A and EUC, they cannot readily be generalized to other clinical populations. Future studies should aim to test whether the BER-5 is applicable and acceptable in other clinical settings and whether it can predict relevant outcomes such as real-time measures of emotion dysregulation or associated problem behaviours. Specifically, it would be interesting to replicate the study within a sample that includes more participants with BPD and/or other personality disorders. In such a study, the BER-5 could be analysed in comparison with dimensional measures of personality pathology such as the PID-5 [29] to examine potential associations with negative affectivity as well as other trait domains.

## Conclusions

The BER-5 is a brief, pragmatic measure of emotion dysregulation with good psychometric properties and might constitute a useful screening tool for clinicians working in specialized health care settings. Future studies should examine its predictive validity in relation to categorical and dimensional personality disorder constructs and, further, study the properties of the scale in different clinical contexts and socio-cultural settings.

## Supplementary Information

Supplementary Material 1.

## Data Availability

A syntax file as well as output from the statistical analyses are included in the supplementary materials and in the manuscript text. When it comes to sharing of data, this is compromised as participants were informed at the signing of consent that the data would be stored in a safe encrypted database only available for researchers associated with the current project.
