# Supplementary material for: Brief, pragmatic measure of emotion dysregulation in young people – a preliminary validation of the BER-5
Source: Borderline Personal Disord Emot Dysregul. 2025 Mar 13;12:8. doi: 10.1186/s40479-025-00285-4 (PMC11905671; doi:10.1186/s40479-025-00285-4)

**SUPPLEMENTARY MATERIALS**

Table S1. Eigen values and explained variance in a principal component analysis of the BER-5

| Component | Total | Initial Eigenvalue: Proportion of variance % | Initial Eigenvalue: Cumulative proportion % |
| --- | --- | --- | --- |
| 1 | 2.97 | 59.48 | 59.48 |
| 2 | 1.02 | 20.37 | 79.85 |
| 3 | .41 | 8.28 | 88.12 |
| 4 | .36 | 7.31 | 95.44 |
| 5 | .22 | 4.55 | 100.00 |

Table S2. Inter-item correlations between BER-5 items at baseline and at the 3.1 years follow-up

| Baseline | Q1_1 | Q2_1 | Q3_1 | Q4_1 | Q5_1 |
| --- | --- | --- | --- | --- | --- |
| Q1_1 | 1.00 |  |  |  |  |
| Q2_1 | 0.32* | 1.00 |  |  |  |
| Q3_1 | 0.40* | 0.35* | 1.00 |  |  |
| Q4_1 | 0.04 | 0.48* | 0.40* | 1.00 |  |
| Q5_1 | 0.22 | 0.40* | 0.35* | 0.53* | 1.00 |
| Follow-up at 3.1 years |  |  |  |  |  |
| Q1_3 | 1.00 |  |  |  |  |
| Q2_3 | 0.27* | 1.00 |  |  |  |
| Q3_3 | 0.47* | 0.17 | 1.00 |  |  |
| Q4_3 | 0.26* | 0.29* | 0.28* | 1.00 |  |
| Q5_3 | 0.39* | 0.09 | 0.66* | 0.28* | 1.00 |

Table S3. Sensitivity and specificity values for ROC analysis of DERS-16 +/- 50 and BER-5.

| Cut-off point | Sensitivity | Specificity | Correctly classified | LR + | LR - |
| --- | --- | --- | --- | --- | --- |
| >=0 | 100 % | 0 % | 26.67 % | 1.00 | - |
| >=1 | 93.75 % | 29.55 % | 46.67 % | 1.33 | 0.21 |
| >=2 | 87.50 % | 45.45 % | 56.67 % | 1.60 | 0.27 |
| >=3 | 81.25 % | 54.55 % | 61.67 % | 1.78 | 0.34 |
| >=4 | 81.25 % | 72.73 % | 75.00 % | 2.97 | 0.25 |
| **>=5** | **81.25 %** | **84.09 %** | **83.33 %** | **5.10** | **0.22** |
| >=6 | 62.50 % | 88.64 % | 81.67 % | 5.5 | 0.42 |
| >=7 | 56.25 % | 93.18 % | 83.33 % | 8.25 | 0.46 |
| >=8 | 18.75 % | 95.45 % | 75.00 % | 4.12 | 0.85 |
| >=9 | 0 % | 97.73 % | 71.67 % | 0.00 | 1.02 |
| >9 | 0 % | 100 % | 73.33 % | - | 1.00 |

Table S4. Sensitivity and specificity values for ROC analysis of DERS-16 +/- 57.5 and BER-5.

| Cut-off point | Sensitivity | Specificity | Correctly classified | LR + | LR - |
| --- | --- | --- | --- | --- | --- |
| >=0 | 100 % | 0 % | 15.00 % | 1.00 | - |
| >=1 | 100 % | 27.45 % | 38.33 % | 1.37 | 0.00 |
| >=2 | 100 % | 43.14 % | 51.67 % | 1.75 | 0.00 |
| >=3 | 88.89 % | 50.98 % | 56.67 % | 1.81 | 0.21 |
| >=4 | 88.89 % | 66.67 % | 70.00 % | 2.66 | 0.16 |
| **>=5** | **88.89 %** | **76.47 %** | **78.33 %** | **3.77** | **0.14** |
| >=6 | 66.67 % | 82.35 % | 80.00 % | 3.77 | 0.40 |
| >=7 | 55.56 % | 86.27 % | 81.67 % | 4.04 | 0.51 |
| >=8 | 33.33 % | 96.08 % | 86.67 % | 8.50 | 0.69 |
| >=9 | 0 % | 98.04 % | 83.33 % | 0.00 | 1.02 |
| >9 | 0 % | 100 % | 85.00 % | - | 1.00 |

Table S5. Sensitivity and specificity of DERS-16 and BPD diagnosis

| Cut-off point | Sensitivity | Specificity | Correctly classified | LR + | LR - |
| --- | --- | --- | --- | --- | --- |
| >=16 | 100 % | 0 % | 1.33 % | 1.00 | - |
| >=18 | 100 % | 1.92 % | 15.00 % | 1.02 | 0.00 |
| >=21 | 100 % | 3.85 % | 16.67 % | 1.04 | 0.00 |
| >=22 | 100 % | 11.54 % | 23.33 % | 1.13 | 0.00 |
| >=23 | 100 % | 13.46 % | 25.00 % | 1.16 | 0.00 |
| >=24 | 100 % | 23.08 % | 33.33 % | 1.30 | 0.00 |
| >=28 | 100 % | 26.92 % | 36.67 % | 1.36 | 0.00 |
| >=31 | 100 % | 28.85 % | 38.33 % | 1.40 | 0.00 |
| >=34 | 100 % | 34.62 % | 43.33 % | 1.52 | 0.00 |
| >=35 | 100 % | 36.54 % | 45.00 % | 1.58 | 0.00 |
| >=37 | 100 % | 38.46 % | 46.67 % | 1.63 | 0.00 |
| >=38 | 100 % | 40.38 % | 48.33 % | 1.68 | 0.00 |
| >=40 | 100 % | 44.23 % | 51.67 % | 1.79 | 0.00 |
| >=41 | 100 % | 50.00 % | 56.67 % | 2.00 | 0.00 |
| >=42 | 100 % | 53.85 % | 60.00 % | 2.17 | 0.00 |
| >=43 | 100 % | 57.69 % | 63.33 % | 2.36 | 0.00 |
| >=44 | 100 % | 61.54 % | 66.67 % | 2.60 | 0.00 |
| >=45 | 100 % | 67.31 % | 71.67 % | 3.06 | 0.00 |
| >=46 | 100 % | 69.23 % | 73.33 % | 3.25 | 0.00 |
| >=48 | 100 % | 73.08 % | 76.67 % | 3.71 | 0.00 |
| >=49 | 87.50 % | 76.92 % | 78.33 % | 3.79 | 0.16 |
| >=51 | 87.50 % | 82.69 % | 83.33 % | 5.06 | 0.15 |
| >=52 | 75.00 % | 84.62 % | 83.33 % | 4.87 | 0.29 |
| >=54 | 75.00 % | 86.54 % | 85.00 % | 5.57 | 0.29 |
| >=55 | 75.00 % | 88.46 % | 86.67 % | 6.50 | 0.28 |
| >=56 | 75.00 % | 90.38 % | 88.33 % | 7.80 | 0.28 |
| >=57 | 75.00 % | 92.31 % | 90.00 % | 9.75 | 0.27 |
| >=58 | 75.00 % | 94.23 % | 91.67 % | 13.00 | 0.26 |
| >=61 | 62.50 % | 94.23 % | 90.00 % | 10.83 | 0.39 |
| >=62 | 50.00 % | 94.23 % | 91.67 % | 8.66 | 0.53 |
| >=63 | 50.00 % | 96.15 % | 90.00 % | 13.00 | 0.52 |
| >=65 | 50.00 % | 98.08 % | 91.67 % | 26.00 | 0.51 |
| >=70 | 37.50 % | 98.08 % | 90.00 % | 19.50 | 0.63 |
| >=71 | 12.50 % | 98.08 % | 86.67 % | 6.50 | 0.89 |
| >=80 | 12.50 % | 100.00 % | 88.33 % | - | 0.87 |
| >80 | 0.00 % | 100.00 % | 86.67 % | - | 1.00 |

**Figures**

Figure S1. ROC analysis of BER-5 compared with DERS-16 dichotomized at 50


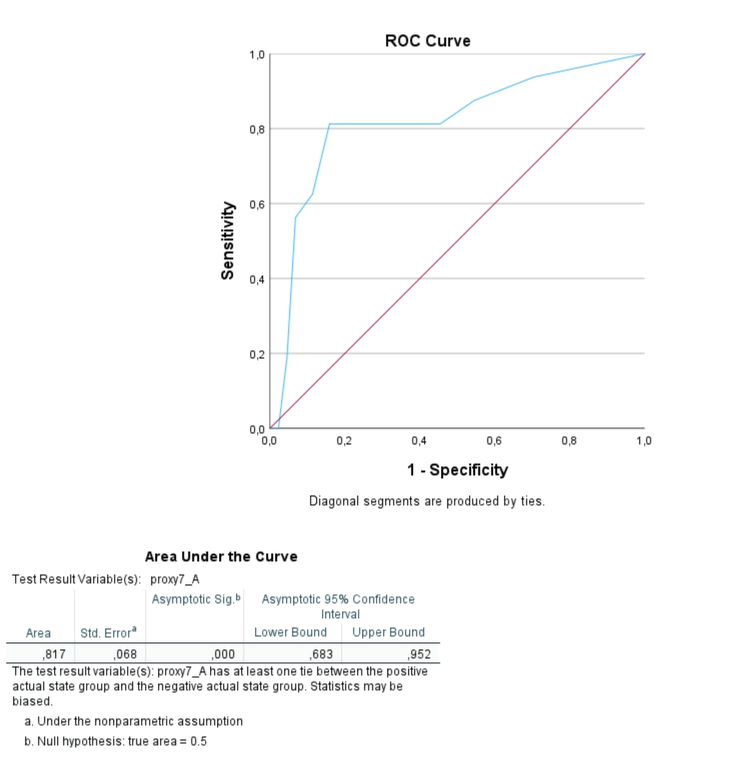


Figure S2. ROC analysis of BER-5 compared with DERS-16 dichotomized at 57.5


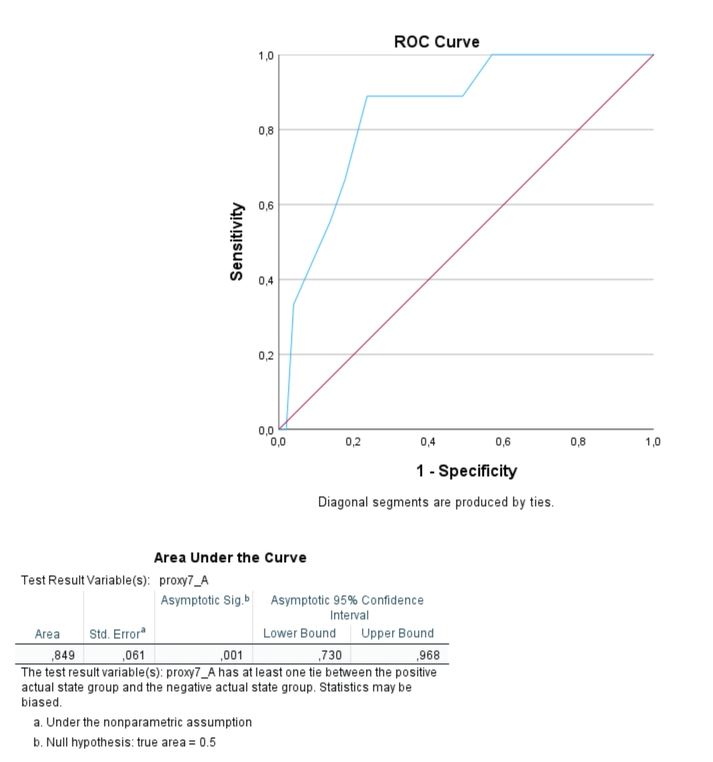


**Syntax for scoring BER-5 (STATA)**

BPD1_6 corresponds to the 6^th^ BPD trait in SCID-II, ‘emotional instability’

**gen** Q1 =.

**replace** Q1 = 0 if BPD1_6==1

**replace** Q1 = 1 if BPD1_6==2

**replace** Q1 = 3 if BPD1_6==3

BPD1_8 corresponds to the 8^th^ BPD trait in SCID-II, ‘intense anger’

**gen** Q2 =.

**replace** Q2 = 0 if BPD1_8==1

**replace** Q2 = 1 if BPD1_8==2

**replace** Q2 = 3 if BPD1_8==3

YSR_87 corresponds to the YSR item ‘emotional instability’

**gen** Q3 = YSR1_87

YSR_95 corresponds to the YSR item ‘emotional instability’

**gen** Q4 = YSR1_95

BSL1 corresponds to the 14^th^ BSL-23 item ‘emotional lability’

**gen** Q5 =.

**replace** Q5 = 1 if BSL1==1

**replace** Q5 = 1 if BSL1==2

**replace** Q5 = 1 if BSL1==3

**replace** Q5 = 2 if BSL1==4

**gen** BER5 = (Q1+Q2+Q3+Q4+Q5)
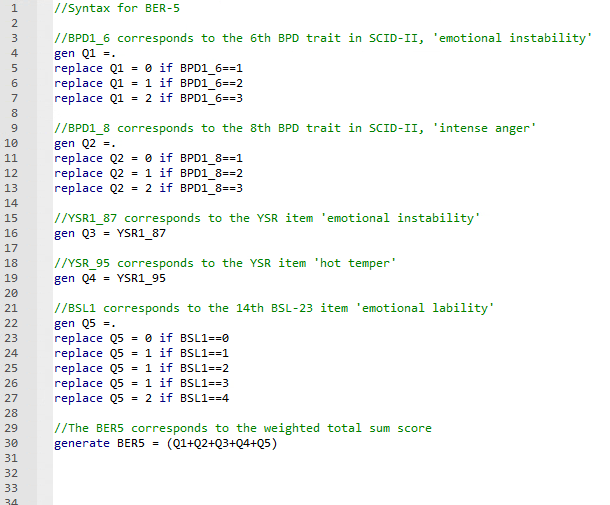

Supplement: Supplementary file 1 — Supplementary Material 1. [file 40479_2025_285_MOESM1_ESM.docx]
